# Comparative Analysis of Gut Microbiota in Humans Living with and Without Companion Animals

**DOI:** 10.3390/life14121621

**Published:** 2024-12-06

**Authors:** Kyung-Hyo Do, Jiwon Park, Nahee Kim, Dahye Ryu, Min-Gyu Kim, Hyunjung Ahn, Hakhyun Kim, Jun-Gi Hwang, Min-Kyu Park, Kwang-Won Seo, Wan-Kyu Lee

**Affiliations:** 1 College of Veterinary Medicine, Chungbuk National University, Cheongju 28644, Republic of Korea; pollic@chungbuk.ac.kr (K.-H.D.);; 2 GutBiomeTech Co., Ltd., Cheongju 28644, Republic of Korea; 3 Department of Clinical Pharmacology and Therapeutics, College of Medicine and Hospital, Chungbuk Natioanl University, Cheongju 28644, Republic of Korea

**Keywords:** intestinal microbiology, environmental health, intestinal bacteria, companion animal

## Abstract

Cohabitation with companion animals (CAs) has been suggested as a significant modifier of gut microbial diversity. This study investigated the influence of cohabitation with CAs on human gut microbiota composition. Using 16S rRNA gene sequencing, we analyzed the gut microbiota of 20 families with CAs (40 adults, 20 children) and 20 families without CAs (40 adults, 20 children). Alpha and beta diversity analyses were performed, and the differentially abundant genera were identified. There were significant differences in beta diversity across the groups (*p*-value = 0.001). The *Bacillota*/*Bacteroidota* ratio was considerably lower in the CAs group (0.67) than in the without-CAs group (1.02). *Prevotellaceae*_UCG-003 (log2 fold change: 7.3; adjusted *p*-value ≤ 0.001), *Ruminococcaceae* (log2 fold change: 6.3; adjusted *p*-value ≤ 0.001), and *Oscillospira* (log2 fold change: 5.1; adjusted *p*-value = 0.012) were elevated in the group cohabiting with CAs, whereas *Megamonas* (with CAs: 3.81%; without CAs: 13.52%) and *Veillonella* (with CAs: 3.77%; without CAs: 6.50%) were more prevalent in the group without CAs. Cohabitation with CAs may positively influence the gut microbiota by promoting the presence of beneficial bacteria and reducing the *Bacillota*/*Bacteroidota* ratio. This study highlights the potential for cohabitation with CAs to promote gut microbial health.

## 1. Introduction

The microbiota, collectively referring to the myriad microorganisms residing in the mammalian gut, has a profound influence on health and disease [1,2]. The presence or augmentation of beneficial bacteria, such as probiotics, is advantageous for the treatment of diseases. However, an increase in certain bacteria within the intestine may contribute to disease onset [3,4]. Therefore, maintaining the diversity and stability of gut microbiota is important for maintaining health [5,6,7].

Recent studies have increasingly highlighted how genetic and various environmental factors influence the composition of the gut microbiota throughout a person’s life [3,4,7,8,9,10,11,12,13,14]. However, no significant differences in microbial community similarity were observed between monozygotic and dizygotic twins [14], suggesting that genetic factors alone do not fully explain the composition of the gut microbiota. In a study conducted on American adolescent twins and their parents, the gut microbiota distribution of adolescents was similar to that of their parents, and there was a high degree of microbial similarity between the parents themselves [15]. This demonstrates that shared environmental or lifestyle factors may influence the composition of gut microbiota.

Previous studies have focused on how various environmental elements can change microbiome composition throughout a person’s life [5,7,16,17]. Although genetic factors play a remarkable role, environmental factors such as cohabitation with companion animals (CAs) have also emerged as key influencers of gut microbiota composition. Among these environmental factors, cohabitation with CAs has become a particularly intriguing area of study [2]. Coelho et al. [18] reported significant similarities between human microbiomes and those of CAs, with growing evidence indicating that the gut microbiota plays a crucial role in managing health and diseases in domestic CAs [6,19,20,21].

However, limited research has explored whether individuals living with CAs harbor distinct gut microbiota compared to those without CAs and how these changes might influence metabolic health. Emerging evidence suggests that gut microbiota diversity and composition are associated with reduced risks of metabolic syndrome, a condition characterized by abdominal obesity, dyslipidemia, hypertension, and impaired insulin sensitivity [22,23]. Understanding the potential role of cohabitation with CAs in shaping gut microbiota to mitigate these risks could have significant implications for public health.

CAs can affect the gut microbiota of caregivers in various ways. Caregivers take pleasure in engaging with their CAs, partaking in close interactions like sharing sleeping spaces, caressing, embracing, and making direct head contact [24]. Caretakers often need to dispose of the feces for CAs, which is a major cause of microbial changes in the environment [24]. Injury to caregivers during interaction with CAs can lead to bacterial infection [24]. Interactions with CAs have been associated with alterations in the gut microbiota composition attributed to the transfer of microbiota and modifications in lifestyle practices [25,26].

Given this background, we hypothesized that individuals cohabiting with CAs harbor a distinct gut microbiota compared to those who do not. In particular, we aimed to explore whether living with CAs contributes to microbial changes that could potentially reduce the risk of metabolic conditions, such as obesity and other markers of metabolic syndrome. In this study, we compared and analyzed the gut microbiome of individuals who have cohabited with CAs for an extended period with those who did not live with CAs. Through this investigation, we sought to provide new insights into the relationship between human–animal interaction and gut microbiota composition.

## 2. Materials and Methods

### 2.1. Study Participants

This study was approved by the Institutional Review Board of Chungbuk National University (approval no.: CBNU-202303-HRBR-0056; approval date: 31 March 2023). Fully informed written consent was obtained from all participants. The written consent that was obtained from all participants included permission for publication. We recruited 60 individuals from 20 families cohabiting with CAs (40 adults and 20 children) and 60 individuals from 20 families without CAs (40 adults and 20 children), ensuring that there were no statistically significant disparities in age and gender between the two groups. Participants cohabiting with CAs were required to have lived with a CA (14 dogs and 7 cats) for more than three years to reduce variability caused by short-term exposure. Participants were recruited from the same urban region in 2023 to minimize geographic and environmental variability between groups. While detailed dietary habits and specific health or mental health data were not collected, all participants were screened to ensure they had no significant underlying health conditions based on their medical history and biochemical test results. Blood was drawn from all individuals to evaluate their complete blood cell counts and conduct biochemical analyses. Stool samples were collected aseptically and anaerobically from each participant and preserved at −70 °C until analyzed.

### 2.2. Sample Collection and DNA Extraction

Participants provided fresh fecal samples in sterile containers with Anaerobic Transport Medium (Cat# AS-911; Anaerobe Systems, Morgan Hill, CA, USA), which were then frozen at −70 °C. From each individual’s fecal sample, 200 mg was used to extract bacterial DNA utilizing the Maxwell RSC Instrument (Promega, Madison, WI, USA) and the Maxwell RSC Fecal Microbiome DNA Kit AS1700 (Promega) following the instructions provided by the manufacturer. Following extraction, DNA quality was evaluated using the Qubit dsDNA HS assay kit (Life Technologies, Gent, Belgium) and measured on a Qubit fluorometer (Thermo Fisher Scientific, Cleveland, OH, USA), in accordance with the manufacturer’s guidelines.

### 2.3. 16S rRNA Gene Sequencing

Following measurement with a Qubit dsDNA HS assay kit on a Qubit fluorometer, 16S V3-V4 amplicon libraries were prepared using the protocol outlined in the Illumina metagenomic sequencing library preparation guide (Part # 15,044,223 Rev. B, Illumina, San Diego, CA, USA). In summary, the V3-V4 regions of the 16S rRNA bacterial gene were amplified utilizing the KAPA HiFi HotStart ReadyMix PCR Kit (KAPA Biosystems, Wilmington, MA, USA) with specified primers (16S rRNA gene-specific sequences are underlined): forward; 5′-TCGTCGGCAGCGTCAGATGTGTATAAGAGACAGCCTACGGGNGGCWGCAG-3′ and reverse; 5′-GTCTCGTGGGCTCGGAGATGTGTATAAGAGACAGGACTACHVGGGTATCTAATCC-3′.

A subsequent polymerase chain reaction (PCR) added index adapters for sample multiplexing, utilizing the Nextera XT Index Kit (Illumina, San Diego, CA, USA). The concentration of each DNA library was measured with a Qubit fluorometer (Thermo Fisher Scientific, Cleveland, OH, USA). Libraries were combined at a 4 nM concentration and denatured using 0.2 N NaOH. The libraries were then diluted to a final concentration of 7 pM, incorporating the phiX control library at 30% (*v*/*v*) of the same concentration for quality control. Sequencing of the library was executed on the MiSeq system following Illumina’s prescribed protocol with the MiSeq Reagent Kit v3 (600 cycles).

### 2.4. Bioinformatic Analysis and Statistical Analysis

For demographic and clinical data analysis, the chi-squared test was used to compare the proportions of male and female participants between groups. For continuous variables (such as hemoglobin, white blood cell count, and biochemical parameters), independent *t*-tests were performed to compare differences between participants living with and without CAs. A *p*-value of <0.05 was considered statistically significant for all tests.

The sequences of the 16S rRNA gene were obtained and analyzed using the QIIME 2 (2023.7) classify-sklearn naïve Bayes classifier protocol. The process included the de-multiplexing and trimming of the reads. The forward and reverse reads were then combined, aligning them with their corresponding samples based on their unique indices. Further trimming was carried out using QIIME to refine the data quality. The sequences underwent a quality check, removing any that fell outside the acceptable range of 20 to 300 nucleotides in length. Additionally, sequences identified as chimeric were discarded. Operational taxonomic units (OTUs) were grouped using the SILVA v138 99% full-length database as a reference. Before proceeding to calculate alpha and beta diversity metrics, the dataset was normalized through rarefaction. The adequacy of the sample size and the prediction of species abundance were assessed with a rarefaction curve analysis.

Species richness and evenness were evaluated by applying the Chao1 alpha diversity index, with consideration given to the depth of sampling. These alpha diversity metrics were derived using QIIME 2 software (version 2023.7). To assess beta diversity, Bray–Curtis dissimilarity measures were utilized, followed by principal coordinate analysis for visualization. Beta diversity differences between groups were statistically tested using PERMANOVA (Permutational Multivariate Analysis of Variance), which evaluates groupwise dissimilarities based on the Bray–Curtis index. The quantification of gut bacteria abundance was conducted using MicrobiomeAnalyst (https://www.microbiomeanalyst.ca/, accessed on 12 December 2023).

Differential abundance of bacterial genera between groups was analyzed using the DESeq2 package [27]. Raw read count data were normalized using the median ratio method, where counts were scaled according to sample-specific size factors. These size factors were determined by calculating the median ratio of observed counts to the geometric mean for each genus. After normalization, the data were exported for further analysis. The Wald test was applied to identify genera with statistically significant changes, and adjusted *p*-values of <0.05 were considered indicative of significance.

The linear discriminant analysis effect size (LEfSE) analysis was performed with a Kruskal–Wallis test (*p*-value ≤ 0.01) for groupwise comparisons and the pairwise Wilcoxon test (*p*-value < 0.01) for comparisons between participants cohabiting with companion animals (with CAs) and those without companion animals (without CAs). The linear discriminant analysis (LDA) scores above 4 were considered significant.

For evaluating microbial relationships within different groups, the SparCC algorithm, which is tailored for compositional data, was utilized [28]. This algorithm applies a log-ratio transformation and iterates to detect pairs of taxa (genera) that significantly deviate from the background correlation pattern. Furthermore, the Sparse Inverse Covariance Estimation for Ecological Association and Statistical Inference (SPIEC-EASI) approach, which employs graphical network models, was used to deduce the overall correlation network in one go [29]. Correlations were only considered significant and visualized if they had an absolute R-correlation value greater than 0.6 and a *p*-value less than 0.05.

## 3. Results

### 3.1. Clinical Data of Participants

The detailed characteristics of all participants, including complete blood cell counts and blood chemical test results, are presented in Table 1 (Additional demographic and antibiotic/probiotic usage information for participants were provided in Supplementary Table S1). There were no significant differences in age or male/female ratio between the groups. There were no significant differences in the levels of total bilirubin, alkaline phosphatase (ALP), aspartate aminotransferase (AST), alanine aminotransferase (ALT), γ-GTP, glucose, total cholesterol, high-density lipoprotein (HDL) cholesterol, low-density lipoprotein (LDL) cholesterol, triglyceride, insulin, hematocrit, mean corpuscular volume (MCV), mean corpuscular hemoglobin (MCH), segmented neutrophils, lymphocytes, monocytes, eosinophils, and basophils between all groups.

The level of hemoglobin was significantly higher in adults cohabiting with CAs (14.1 ± 1.6 g/dL), compared to adults without CAs (13.5 ± 1.6 g/dL) (*p*-value: 0.049). The level of white blood cells (*p*-value: 0.033), platelets (*p*-value: 0.039), and the mean corpuscular hemoglobin concentration (MCHC) (*p*-value: 0.029), in groups cohabiting with CAs (7.6 ± 1.8 × 10^3^/μL, 311.5 ± 64.6 × 10^3^/μL, and 33.1 ± 1.0 g/dL, respectively), was significantly higher than in groups without CAs (6.9 ± 1.5 × 10^3^/μL, 284.2 ± 72.4 × 10^3^/μL, and 32.6 ± 1.3 g/dL, respectively). Although there were significant differences in the clinical data between the cohorts, the clinical data of all participants were within the normal reference ranges.

### 3.2. Difference in the Gut Microbiota: Alpha Diversity and Beta Diversity

**Figure 1:**
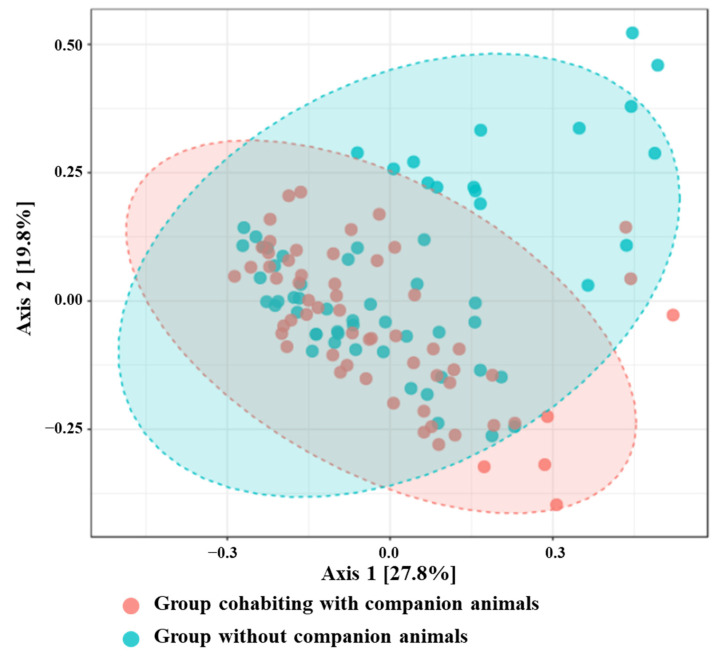
Principal Coordinate Analysis of the Bray–Curtis dissimilarity index. Operational taxonomic units present in <0.1% relative abundance in any sample have been removed. The ellipses represent the 95% confidence intervals for each group.

To assess the degree of genus overlap between groups cohabiting with CAs and those without CAs, we calculated the beta diversity using the Bray–Curtis index (Figure 1). The X- and Y-axes denote the selected spindles, with the percentages reflecting the calculated differences in sample composition. The measurements on the X- and Y-axes represent relative distances that do not hold practical importance. A smaller gap between two points on the sample indicates greater similarity in their species makeup. We found that the microflora structure of the group cohabiting with CAs was significantly different from that of the group without CAs (*p*-value = 0.001).

**Figure 2:**
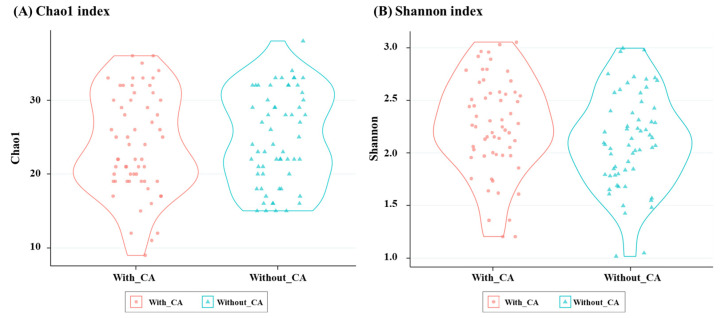
Violin plots of the Chao1 (**A**) and Shannon (**B**) alpha diversity metrics in the groups cohabiting with companion animals and without companion animals. With CAs: group cohabiting with companion animals; without CAs: group without companion animals.

The alpha diversity of the gut microbiome at the genus level, as represented by the Chao1 and Shannon indices, is shown in Figure 2. For the Chao1 index, the overall Chao1 value was 24.3 ± 6.4 (range, 9–37), with the group cohabiting with CAs showing a value of 23.9 ± 6.7 (range, 9–37) and the group without CAs showing a value of 24.7 ± 6.1 (range, 14–35). For the Shannon index, the overall mean Shannon value was 2.2 ± 0.5 (range, 1.0–3.1), with the group cohabiting with CAs having a mean value of 2.2 ± 0.5 (range, 1.2–3.0) and the group without CAs having a mean value of 2.1 ± 0.4 (range, 1.0–3.1). No significant differences were observed between the groups for either the Chao1 or Shannon indices (Chao1, *p* = 0.47; Shannon, *p* = 0.13).

### 3.3. Sharing/Transfer of the Gut Microbiota in the Same Environment

**Figure 3:**
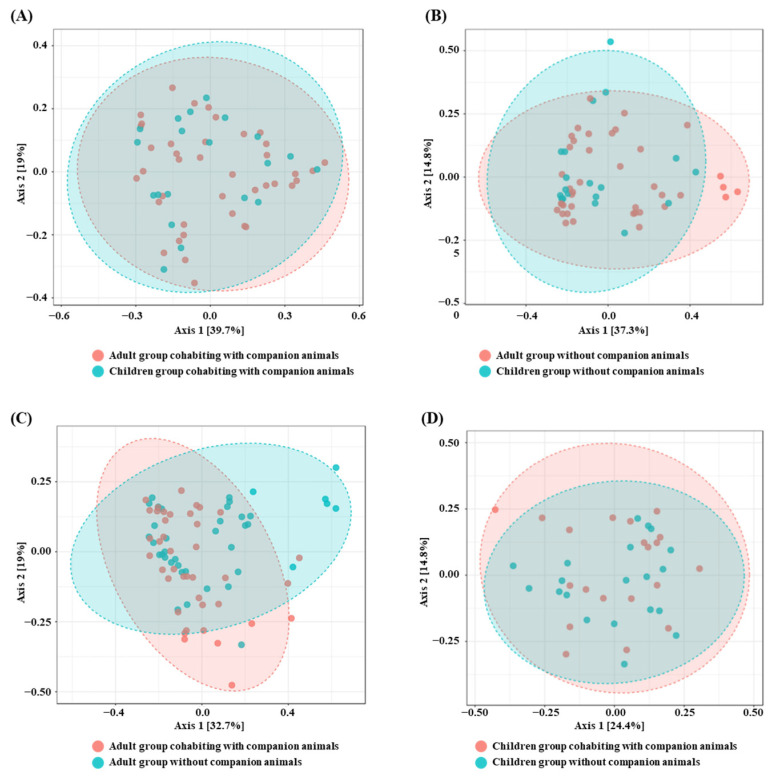
Beta diversity analyses of the gut microbiota in children and adults cohabiting with companion animals (CAs) and without companion animals. The ellipses represent the 95% confidence intervals for each group. (**A**) Beta diversity analysis comparing the adult group cohabiting with CAs and the children group cohabiting with CAs. (**B**) Beta diversity analysis comparing the adult group without CAs and the children group without CAs. (**C**) Beta diversity analysis comparing the adult group cohabiting with CAs and the adult group without CAs. (**D**) Beta diversity analysis comparing the children group cohabiting with CAs and the children group without CAs. Each plot displays distinct clustering patterns, illustrating the impact of cohabitation with CAs on gut microbiota composition. Red and blue colors represent participants without and with CAs, respectively.

A comparison between the gut microbiota of adults and children at the genus level, living in the same environment, is shown in Figure 3. Beta diversity analysis revealed no significant differences between adults and children cohabiting with CAs (*p*-value = 0.36) or between adults and children without CAs (*p*-value = 0.22), indicating that the gut microbiota within the same environment tends to be similar. A significant difference in beta diversity was observed between adults with and without CAs (*p*-value = 0.03), whereas no significant difference was observed between children with and without CAs (*p*-value = 0.71).

### 3.4. Overall Composition of the Intestinal Microbiota

**Figure 4:**
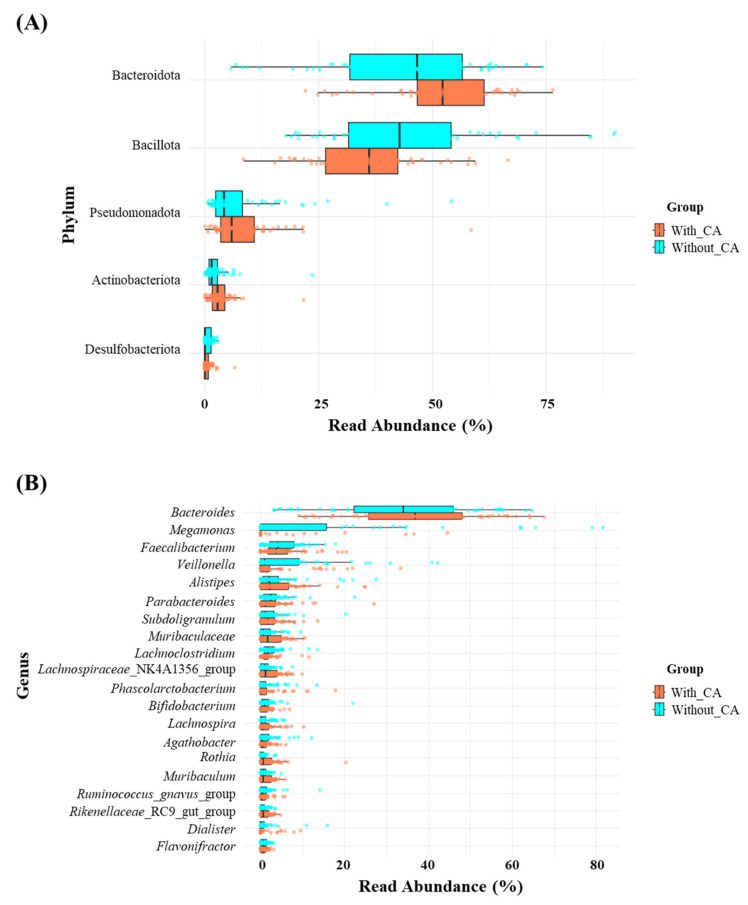
Boxplot of the relative read abundances of (**A**) the top 5 phyla and (**B**) the top 20 genera present in the gut microbiota in the groups cohabiting with companion animals and without companion animals. With CAs: group cohabiting with companion animals; without CAs: group without companion animals.

To visualize the distribution of the intestinal microbiota in participants cohabiting with CAs and without CAs at the phylum and genus levels, a stacked bar figure was prepared (Figure 4). The primary phyla observed in the groups cohabiting with CAs and those without CAs included *Bacteroidota* (with CAs: 52.53%; without CAs: 44.76%), *Bacillota* (with CAs: 35.79%; without CAs: 44.83%), *Pseudomonadota* (with CAs: 7.76%; without CAs: 7.46%), *Actinobacteriota* (with CAs: 3.44%; without CAs: 2.32%), and *Desulfobacterota* (with CAs: 0.49%; without CAs: 0.64%). The *Bacillota*/*Bacteroidota* ratio was considerably high in the group without CAs (with CAs: 0.67; without CAs: 1.02).

At the genus level, the primary genera observed in the groups cohabiting with CAs and those without CAs included *Bacteroides* (with CAs: 37.72%; without CAs: 33.28%), *Megamonas* (with CAs: 3.81%; without CAs: 13.52%), *Faecalibacterium* (with CAs: 4.53%; without CAs: 4.51%), *Veillonella* (with CAs: 3.77%; without CAs: 6.50%), and *Alistipes* (with CAs: 3.65%; without CAs: 3.69%).

The relative abundances of *Megamonas* (with CAs: 3.81%; without CAs: 13.52%) and *Veillonella* (with CAs: 3.77%; without CAs: 6.50%) were considerably higher in the group without CAs than in the groups cohabiting with them. The analysis of samples showed large inter-individual variations in the composition of the intestinal microflora at the phylum and genus levels among both groups.

### 3.5. Differences in the Gut Microbiota

**Figure 5:**
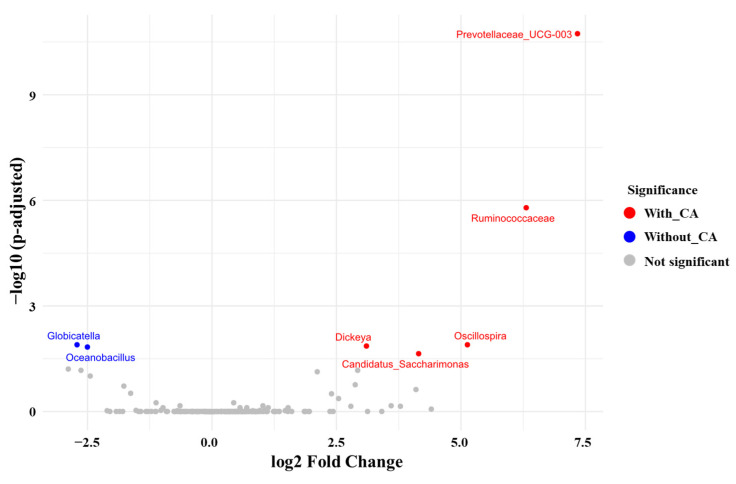
Volcano plot of differentially abundant genera in gut microbiota between groups cohabiting with and without companion animals. The volcano plot illustrates the differential abundance of gut microbial genera between the two groups: individuals cohabiting with companion animals (with CAs) and those without (without CAs). The x-axis represents the log2 fold change, indicating the magnitude of abundance differences, while the y-axis shows the −log10 adjusted *p*-value, reflecting the statistical significance. Genera in red are significantly more abundant in the “With CAs” group, while genera in blue are more abundant in the “Without CAs” group (adjusted *p* < 0.05). Non-significant genera are shown in gray.

To analyze the differential abundance of taxa between the two experimental groups, we used DESeq2 to assess notable genus-level changes in microbial composition and presented the results using a volcano plot (Figure 5).

A volcano plot revealed significantly elevated levels of *Prevotellaceae*_UCG-003 (log2 fold change: 7.3; adjusted *p*-value ≤ 0.001), *Ruminococcaceae* (log2 fold change: 6.3; adjusted *p*-value ≤ 0.001), *Oscillospira* (log2 fold change: 5.1; adjusted *p*-value = 0.012), *Dickeya* (log2 fold change: 3.1; adjusted *p*-value = 0.013), and *Candidatus_Saccharimonas* (log2 fold change: 4.2; adjusted *p*-value = 0.023) in the group with CAs. In contrast, *Globicatella* (log2 fold change: –2.7; adjusted *p*-value = 0.012) and *Oceanobacillus* (log2 fold change: –2.5; adjusted *p*-value = 0.015) were significantly more prevalent in the group without CAs.

**Figure 6:**
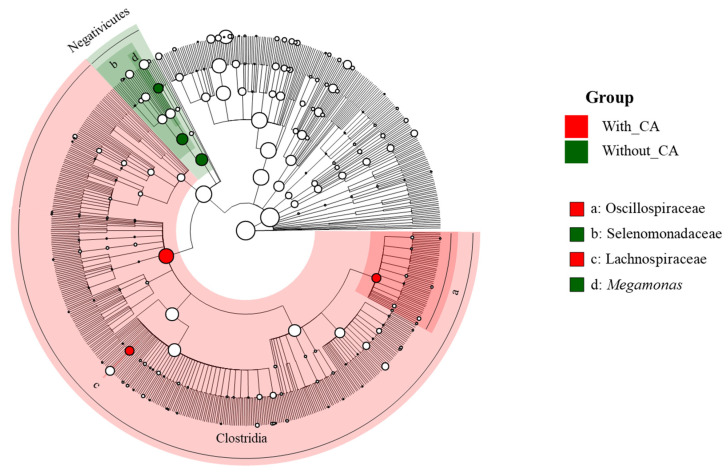
LEfSe analysis of gut microbiota in individuals cohabiting with and without companion animals. With CA: group cohabiting with companion animals; without CAs: group without companion animals. The cladogram represents phylogenetic levels, with each circle indicating a different taxonomic rank. Red regions highlight taxa significantly enriched in the with-CAs group, while green regions highlight taxa enriched in the without-CAs group. Differentially abundant taxa are annotated on the right side of the cladogram.

To further investigate the variations among the intestinal microbial communities of individuals cohabiting with and without companion animals, a LEfSe was performed with an LDA cutoff of 4.0 (Figure 6). A cladogram generated based on the LDA effect size showed that the taxa enriched in the with-CAs group primarily belonged to the class Clostridia, including significant families such as *Oscillospiraceae* (LDA = 4.21, *p*-value = 0.0009) and *Lachnospiraceae* (LDA = 4.17, *p*-value = 0.0078). Conversely, the taxa enriched in the without-CAs group were primarily classified within the class *Negativicutes*, including the family *Selenomonadaceae* (LDA = 4.81, *p*-value = 0.0005) and the genus *Megamonas* (LDA = 4.80, *p*-value = 0.0014).

### 3.6. Microbial Interactions: Correlation Analysis

**Figure 7:**
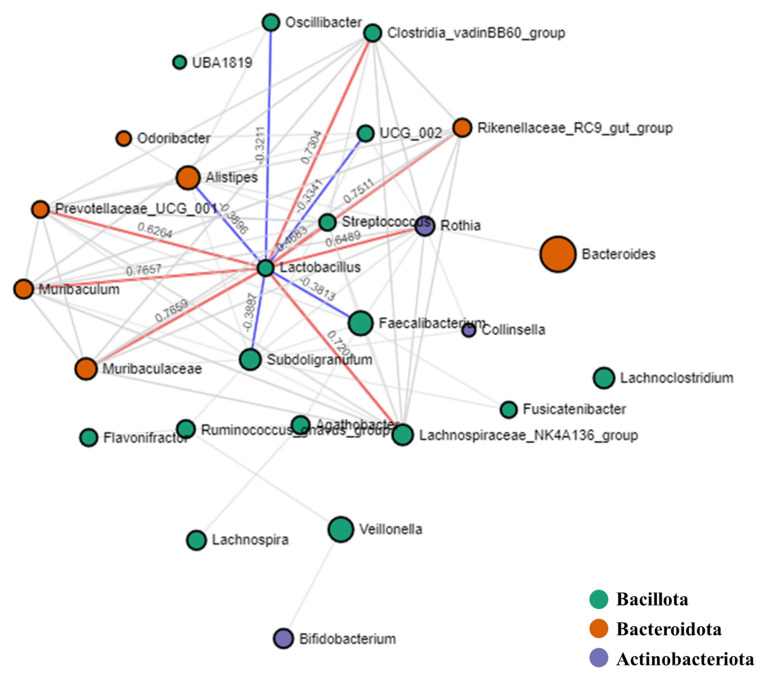
Correlation network analysis. Correlation network maps were generated using the SparCC approach. Each node symbolizes a genus, with the node’s size reflecting the median relative abundance of that genus. The connections, or edges, between nodes demonstrate correlations among genera. Solid lines depict the correlation values for *Lactobacillus*, with positive correlations (co-occurrence) shown in red and negative correlations (mutual exclusion) in blue.

Network analysis of the microbial interactions using SparCC permutations revealed 152 interactions among 26 taxa, of which 108 were positive and 44 were negative (Figure 7). All identified taxa belonged to three major phyla: *Bacillota* (16 genera), *Bacteroidota* (7 genera), and Actinobacteria (3 genera). The genus *Lactobacillus* was positively correlated with *Prevotellaceae*_UCG_001 (correlation, 0.6264) and negatively correlated with *Alistipes* (correlation, −0.3896).

## 4. Discussion

This study examined how cohabiting with companion animals (CAs) influences gut microbiota in adults. The findings suggest that cohabitation with CAs influences microbial community structure and may affect human health in various ways.

Both groups showed no signs of underlying health conditions that could influence gut microbiota composition. While no significant differences were observed in the richness and diversity (alpha diversity) of the gut microbiota, there was a marked difference in its composition (beta diversity) between the groups, with several genera showing differential abundance. Differences in beta diversity revealed substantial variations in the gut microbiota composition between the groups, indicating that cohabitation with CAs may alter the microbial structure [2,7].

No significant beta diversity differences were found between adults and children within each group; however, significant differences were observed between the same age groups in different environments (e.g., adults with CAs vs. adults without CAs). This is in accordance with other past studies, suggesting that the groups that share the same environmental factors have similar gut microbiota [5,17]. This similarity in gut microbiota may be influenced by shared environmental and lifestyle factors [5,17]. Environmental factors, such as shared living spaces and dietary habits, significantly shape gut microbiota similarity [5]. Environmental overlap, such as shared living spaces and similar dietary regimens, plays a pivotal role in shaping the similarities observed in microbiome composition between groups [2]. Previous studies have shown that the gut microbiome undergoes constant changes in composition over a lifetime [5]; the gut microbiome begins to reflect the adult gut microbiome by 3 or 4 years of age [15,17]. In the current study, we found that adults in different cohorts (with and without CAs) had markedly different gut microbiota; however, there was no significant difference between children in the different groups. This difference may be due to adults having frequent contact with CAs, such as handling their care, compared to children. Adults, usually as caretakers of CAs, need to dispose of the feces of their CAs, which is one of the major causes of changes in the gut microbiota [24], whereas children do not.

In healthy adult humans, the predominant gut microbiota encompasses intestinal bacteria from both the *Bacillota* and *Bacteroidota* (previously known as Bacteroidetes) phyla [22]. *Bacillota* efficiently extract energy from food by fermenting dietary fibers into short-chain fatty acids, supporting both gut health and energy metabolism. *Bacteroidota* break down complex carbohydrates, proteins, and fats, simultaneously aiding immune regulation and preventing harmful pathogen colonization [22]. Ley et al. [9] first reported changes in the primary intestinal phyla, *Bacillota* (previously known as Firmicutes) and *Bacteroidota*, in obese animals. Ley et al. [9] reported that a higher *Bacillota*/*Bacteroidota* ratio in animal models is associated with obesity, while a lower ratio has been linked to improved metabolic health and immune function. A decreased prevalence of *Bacillota*/*Bacteroidota* has been linked to lean body types, youthfulness, cardiovascular well-being, and a well-regulated immune system and is typically viewed as advantageous for health [22]. In this study, the lower *Bacillota*/*Bacteroidota* ratio observed in individuals cohabiting with CAs suggests a potential metabolic benefit. In this study, we observed that the *Bacillota*/*Bacteroidota* ratio was higher in individuals without CAs (1.02) compared to those with CAs (0.67), a pattern linked to metabolic health. This lower ratio in individuals cohabiting with CAs suggests a potential metabolic benefit. However, the relationship remains unclear due to confounding factors such as diet, lifestyle, and environmental exposure. Further controlled studies are required to elucidate the precise impact of cohabiting with CAs on gut microbiota composition.

In the present study, distinct differences in gut microbial composition were observed between individuals cohabiting with companion animals (CAs) and those without. We found that *Megamonas* and *Veillonella* were significantly abundant in individuals without CAs. LEfSe analysis further supported this finding, showing a significant enrichment of *Megamonas* in the without-CAs group. *Megamonas*, which is involved in carbohydrate fermentation, may be associated with a diet high in processed foods and reduced physical activity in the group without CAs [23]. Similarly, *Veillonella*, known for metabolizing lactate, was more abundant in the same group, possibly because of metabolic imbalances resulting from less physical exercise [30]. Additionally, *Selenomonadaceae*, another taxon enriched in the without-CAs group, is known for its role in carbohydrate metabolism [31]. Its increased abundance may be reflective of dietary patterns higher in processed carbohydrates among individuals without CAs. Regular activities, such as walking or playing with CAs, in the group cohabiting with CAs may contribute to a more balanced gut microbiota, highlighting the beneficial influence of regular exercise on microbial health [2]. Conversely, *Prevotellaceae*_UCG-003, *Ruminococcaceae*, *Oscillospira*, and *Lachnospiraceae* were substantially elevated in individuals cohabiting with CAs. These genera, associated with fiber digestion and short-chain fatty acid production, contribute to gut health and metabolic balance [2,16,19]. Lifestyle factors such as high fiber intake and increased physical activity, commonly linked to owning CAs, may promote a more beneficial gut microbial composition by fostering these genera [2,16,19].

Restoring a balanced gut microbiota is essential for intestinal health, supporting digestive function, immune regulation, and pathogen defense. Normalizing dysbiosis also contributes to metabolic health and mental well-being, highlighting its importance in maintaining a resilient intestinal environment [7]. *Lactobacillus* and *Prevotellaceae* play vital roles in supporting intestinal health, with *Lactobacillus* promoting a balanced microbial environment through its probiotic properties [32] and *Prevotellaceae* aiding in dietary fiber fermentation to produce anti-inflammatory short-chain fatty acids [8]. Interestingly, we found that *Lactobacillus* was positively correlated with *Prevotellaceae* but negatively correlated with *Alistipes*. This suggests a possible mechanism by which *Lactobacillus* may create an unfavorable environment for *Alistipes*, potentially through pH alteration via lactic acid secretion, which could lead to reduced *Alistipes* abundance. However, further studies are required to confirm this association [32]. The intricate associations identified emphasize the need for further research to elucidate the precise mechanisms by which *Lactobacillus* contributes to health.

The dominant gut microbiota phyla in dogs, such as *Bacillota* and *Bacteroidota*, overlap with those in humans, suggesting potential microbial exchange through close contact [18]. Moreover, previous studies have shown that humans can acquire infections from their CAs [33,34]. These infections are commonly spread through scratches or bites, with secretions from mucous membranes serving as the primary means of transmission [35]. Research indicates that close interactions between humans and CAs encompass activities such as joint walks, petting the head, bathing the dog, cleaning up after the dog, and cohabiting [24]. Therefore, bacteria in the feces of Cas, as well as in the skin, saliva, and urine of Cas, could be important factors affecting the human gut microbiota.

This study has several limitations that should be acknowledged. First, we only analyzed the bacteria in the feces of companion animals (CAs) and humans. To better understand the effects of cohabitation with CAs on human gut microbiota, other biological samples, such as skin, saliva, and urine, should also be considered in future studies. Second, the study was limited by its strict inclusion criteria, which resulted in the exclusion of many participants and a relatively small sample size. This limited the comprehensiveness of the findings and may reduce the generalizability of the results. Third, the cross-sectional nature of the study makes it difficult to establish causal relationships between cohabitation with CAs and changes in human gut microbiota. Finally, although participants were screened for general health conditions, data on potential confounding factors, such as dietary habits and lifestyle differences, were not collected, which may have influenced gut microbiota composition.

Addressing these limitations will require future research that incorporates a larger and more diverse sample size, longitudinal designs, and comprehensive data collection on potential confounders. Additionally, including analyses of various biological samples beyond feces will help provide a more holistic understanding of the interactions between CAs and human microbiota.

## 5. Conclusions

This study demonstrated significant differences in the gut microbiota composition (beta diversity) between individuals cohabiting with companion animals (CAs) and those without. While alpha diversity showed no significant changes, specific genera, such as *Megamonas* and *Veillonella*, were more abundant in individuals without CAs, whereas beneficial bacteria like *Lactobacillus* were elevated in those cohabiting with CAs. These findings suggest that cohabitation with CAs may positively influence gut microbiota by lowering the *Bacillota*/*Bacteroidota* ratio, a marker associated with metabolic and immune health.

These results highlight the potential role of companion animals in promoting gut health and their broader implications for metabolic balance and disease prevention. However, further studies with larger and more diverse cohorts are needed to validate these findings and to explore the impact of additional factors, such as diet, lifestyle, and pet demographics. Longitudinal research could also help clarify the causal relationships between cohabitation with CAs and changes in gut microbiota.

This study underscores the importance of the human–animal–environment connection within the framework of One Health, emphasizing the need for integrative approaches to understanding and improving human health.

## Figures and Tables

**Table 1:** Clinical data of participants cohabiting with companion animals and participants without companion animals.

Variables	Participants Cohabiting with Companion Animals			Participants Without Companion Animals		
	Adult (n = 40)	Children (n = 20)	Sub-Total (n = 60)	Adult (n = 40)	Children (n = 20)	Sub-Total (n = 60)
Age	41.10 ± 5.60	9.55 ± 2.91	30.58 ± 15.76	42.63 ± 3.99	9.90 ± 2.05	31.72 ± 15.93
Male/Female	20/20	11/9	31/29	20/20	9/11	29/31
Total Bilirubin (mg/dL)	0.5 ± 0.3	0.3 ± 0.1	0.4 ± 0.3	0.5 ± 0.3	0.3 ± 0.1	0.4 ± 0.3
ALP ^(1)^ (U/L)	69.5 ± 21.2	282.2 ± 73.0	140.4 ± 110.6	69.6 ± 17.4	275.1 ± 65.7	138.1 ± 105.5
AST ^(2)^ (U/L)	21.8 ± 9.0	27.8 ± 5.2	23.8 ± 8.4	22.1 ± 15.6	24.6 ± 5.5	22.9 ± 13.1
ALT ^(3)^ (U/L)	25.4 ± 27.3	18.5 ± 21.0	23.1 ± 25.4	22.6 ± 23.5	14.3 ± 6.0	19.8 ± 19.8
γ- GTP ^(4)^ (U/L)	31.0 ± 22.6	14.3 ± 8.2	25.4 ± 20.5	28.0 ± 21.0	13.0 ± 4.0	23.0 ± 18.6
Glucose (mg/dL)	100.6 ± 22.6	90.1 ± 11.6	97.1 ± 20.2	94.9 ± 17.7	86.0 ± 11.4	92.0 ± 16.4
Total Cholesterol (mg/dL)	201.0 ± 41.2	168.6 ± 28.3	190.2 ± 40.3	186.8 ± 35.3	163.9 ± 28.6	179.1 ± 34.8
Free Fatty Acid (μ Eq/L)	392.0 ± 221.5	386.9 ± 189.0	390.3 ± 209.6	369.9 ± 253.2	407.1 ± 229.3	382.3 ± 244.2
HDL ^(5)^ Cholesterol (mg/dL)	56.7 ± 13.5	56.8 ± 9.5	56.8 ± 12.2	58.9 ± 16.6	54.6 ± 11.2	60.8 ± 15.2
LDL ^(6)^ Cholesterol (mg/dL)	119.3 ± 37.5	94.8 ± 23.4	111.1 ± 35.3	107.5 ± 31.5	89.4 ± 26.6	101.5 ± 30.9
Triglyceride (mg/dL)	210.7 ± 167.2	133.5 ± 93.0	185.0 ± 150.4	193.4 ± 152.4	95.0 ± 45.1	160.6 ± 134.9
Insulin (μ IU/mL)	30.1 ± 33.4	39.5 ± 78.0	33.2 ± 52.1	24.3 ± 22.1	18.2 ± 18.7	22.3 ± 21.1
White Blood Cells (×10^3^/μL) ^‡^	7.4 ± 1.8	8.1 ± 1.6	7.6 ± 1.8 ^‡^	6.6 ± 1.6	7.5 ± 1.2	6.9 ± 1.5 ^‡^
Red Blood Cells (×10^6^/μL)	4.6 ± 0.6	4.5 ± 0.4	4.6 ± 0.5	4.5 ± 0.5	4.5 ± 0.3	4.5 ± 0.4
Hemoglobin (g/dL) ^†^	14.1 ± 1.6 ^†^	13.0 ± 1.1	13.8 ± 1.6	13.5 ± 1.6 ^†^	12.9 ± 0.7	13.3 ± 1.4
Hematocrit (%)	42.8 ± 4.4	39.0 ± 3.2	41.6 ± 4.4	41.5 ± 4.3	39.0 ± 3.0	40.7 ± 4.1
Platelet (×10^3^/μL) ^‡^	295. ± 62.1	345.9 ± 57.2	311.5 ± 64.6 ^‡^	265.1 ± 70.5	322.3 ± 61.8	284.2 ± 72.4 ^‡^
MCV ^(7)^ (fL)	92.8 ± 4.7	86.0 ± 3.7	90.6 ± 5.5	92.1 ± 5.4	86.1 ± 3.3	90.1 ± 5.6
MCH ^(8)^ (pg)	30.6 ± 1.6	28.6 ± 1.3	29.9 ± 1.8	29.8 ± 2.4	28.4 ± 1.0	29.3 ± 2.1
MCHC ^(9)^ (g/dL) ^‡^	32.9 ± 1.0	33.3 ± 0.9	33.1 ± 1.0 ^‡^	32.4 ± 1.3	33.0 ± 1.0	32.6 ± 1.3 ^‡^
Segmented Neutrophil (%)	58.0 ± 6.8	49.8 ± 10.5	55.4 ± 9.0	55.3 ± 8.4	47.8 ± 8.4	52.8 ± 9.0
Lymphocyte (%)	31.6 ± 6.0	38.3 ± 8.7	33.7 ± 7.6	34.0 ± 7.9	42.1 ± 8.2	36.7 ± 8.8
Monocyte (%)	7.5 ± 1.7	6.9 ± 1.4	7.3 ± 1.6	7.2 ± 1.6	6.6 ± 1.3	7.0 ± 1.5
Eosinophil (%)	2.3 ± 1.3	4.3 ± 3.3	2.9 ± 2.3	2.9 ± 2.1	3.1 ± 2.4	2.9 ± 2.2
Basophil (%)	0.7 ± 0.3	0.7 ± 0.3	0.7 ± 0.3	0.7 ± 0.3	0.5 ± 0.2	0.6 ± 0.3

All data are expressed as means ± standard deviation. ^†^ indicates significant differences in adults of groups cohabiting with companion animals and participants without companion animals. ^‡^ indicates significant differences in humans (adults and children) between participants cohabiting with companion animals and participants without companion animals. There were no significant differences in the number of children cohabiting with companion animals and those that did not; ^(1)^ ALP: alkaline phosphatase, ^(2)^ AST: aspartate aminotransferase, ^(3)^ ALT: alanine aminotransferase, ^(4)^ GTP: glutamyl transpeptidase, ^(5)^ HDL: high-density lipoprotein; ^(6)^ LDL: low-density lipoprotein, ^(7)^ MCV: mean corpuscular volume, ^(8)^ MCH: mean corpuscular hemoglobin, ^(9)^ MCHC: mean corpuscular hemoglobin concentration.

## Data Availability

The datasets generated and/or analyzed during the current study are available from the corresponding author on reasonable request.

## Supplementary Materials

The following supporting information can be downloaded at: https://www.mdpi.com/article/10.3390/life14121621/s1, Table S1: Demographic and antibiotic/probiotic use information of participants cohabiting with companion animals and participants without companion animals.
